# Assessment of the longitudinal humoral response in non-hospitalized SARS-CoV-2-positive individuals at decentralized sites: Outcomes and concordance

**DOI:** 10.3389/fimmu.2022.1052424

**Published:** 2023-01-20

**Authors:** Abdelhadi Djaïleb, Étienne Lavallée, Megan-Faye Parker, Marie-Pierre Cayer, Florence Desautels, Marie Joëlle de Grandmont, Matthew Stuible, Christian Gervais, Yves Durocher, Sylvie Trottier, Denis Boudreau, Jean-Francois Masson, Danny Brouard, Joelle N. Pelletier

**Affiliations:** ^1^ Département de Chimie, Université de Montréal, Montréal, QC, Canada; ^2^ PROTEO, Regroupement Québécois de Recherche sur la Fonction, l’Ingénierie et les Applications des Protéines, Québec, QC, Canada; ^3^ Centre en Chimie Verte et Catalyse, Université de Montréal, Montréal, QC, Canada; ^4^ Départment de Biochimie et Médecine Moléculaire, Université de Montréal, Montréal, QC, Canada; ^5^ Héma‐Québec, Affaires Médicales et Innovation, Québec, QC, Canada; ^6^ Mammalian Cell Expression, Human Health Therapeutics Research Centre, National Research Council Canada, Montréal, QC, Canada; ^7^ Centre de Recherche du Centre Hospitalier Universitaire de Québec, Université Laval, Québec, QC, Canada; ^8^ Département de Microbiologie-Infectiologie et d’Immunologie, Université Laval, Québec, QC, Canada; ^9^ Départment de Chimie, Université Laval, Québec, QC, Canada; ^10^ Centre d’Optique, Photonique et Laser, Université Laval, Québec, QC, Canada; ^11^ Centre Québécois sur les Matériaux Fonctionnels, Montréal, QC, Canada; ^12^ Centre Interdisciplinaire de Recherche sur le Cerveau et l’Apprentissage, Université de Montréal, Montréal, QC, Canada

**Keywords:** seroconversion, longitudinal study, pandemic testing, variants of concern, ELISA

## Abstract

**Introduction:**

Early in the COVID-19 pandemic, reagent availability was not uniform, and infrastructure had to be urgently adapted to undertake COVID-19 surveillance.

**Methods:**

Before the validation of centralized testing, two enzyme-linked immunosorbent assays (ELISA) were established independently at two decentralized sites using different reagents and instrumentation. We compared the results of these assays to assess the longitudinal humoral response of SARS-CoV-2-positive (i.e., PCR-confirmed), non-hospitalized individuals with mild to moderate symptoms, who had contracted SARSCoV-2 prior to the appearance of variants of concern in Québec, Canada.

**Results:**

The two assays exhibited a high degree of concordance to identify seropositive individuals, thus validating the robustness of the methods. The results also confirmed that serum immunoglobulins persist ≥ 6 months post-infection among non-hospitalized adults and that the antibodies elicited by infection cross-reacted with the antigens from P.1 (Gamma) and B.1.617.2 (Delta) variants of concern.

**Discussion:**

Together, these results demonstrate that immune surveillance assays can be rapidly and reliably established when centralized testing is not available or not yet validated, allowing for robust immune surveillance.

## 1 Introduction

COVID-19 is a respiratory illness caused by SARS-CoV-2 (1), a virus that appeared in late 2019 in Wuhan, China (2). This virus, likely of zoonotic origin (3), rapidly spread worldwide and was declared a global pandemic by the World Health Organization (WHO) on March 11th, 2020 (4). Whereas some people developed serious complications (e.g., pneumonia, blood clotting or respiratory distress) requiring hospitalization (5), at least 95% of people infected by SARS-CoV-2 in Canada (6) [or in the province of Québec (7)] were not hospitalized and generally exhibited mild, moderate, or no symptoms (8, 9).

Amid an outbreak of a new infectious disease with serious public health consequences, protocols must be rapidly and reliably implemented by non-specialized and resource-limited laboratories to detect the various stages of the infection and to study immune response in local settings. Such protocols can be established for population-level surveillance and research support rather than for medical diagnostics, which require approval by regulatory agencies. This was clearly exemplified in the early stages of the SARS-CoV-2 pandemic, as countries needed to provide surveillance protocols rapidly in a multitude of research centers.

Notably, these centers often used reagents from different sources and relied on pre-existing (and often non-optimal) instrumentation already at their disposal; they also faced production and transportation logistic challenges, adopting materials that were later replaced by others. Early in the COVID-19 pandemic, some evocative examples included limited access to disease-specific biological materials that were not fully characterized and had never been produced (e.g., antigens, antibodies) and adaptation to alternate types of disposable plasticware amid worldwide shortages. In addition, unlike centralized laboratories (which were later designated as the main testing sites for SARS-CoV-2 infection and immunity), decentralized laboratories do not generally have the advantage of large-scale automation. Even when centralized testing is established, decentralized laboratories are essential as they provide more rapid test results (i.e., no delays due to shipment or mass processing). As they do not require the execution of strict validation protocols, they offer the flexibility required to support diverse, long-term research projects.

Based on early reports of enzyme-linked immunosorbent assays (ELISA) that target SARS-CoV-2 antigens, a team at University of Montreal (UdeM site) developed and validated an ‘in-house’ ELISA to detect spike- and nucleocapsid-specific IgGs in human serum, plasma and dried blood spots during the first wave of infections in Canada (10). In parallel, a team at Héma-Québec (HQ site; the blood bank of the province of Québec) developed and validated their own ‘in-house’ ELISA assay adapted from a recently described protocol (11–13). Although generally similar, these assays use different reagents and instrumentation, as they were urgently and independently developed by different laboratories.

Here, we investigate the humoral response to spike and nucleocapsid antigens elicited in 81 non-hospitalized, SARS-CoV-2-positive (i.e., PCR-confirmed) adults who exhibited mild to moderate COVID-19 symptoms. Our objective was to extend immunological surveillance beyond the severely ill and hospitalized patients and understand the persistence of the humoral response following infection by SARS-CoV-2. Considering the recruitment period for this study and the SARS-CoV-2 phylogeny mapped in that region, all volunteers likely contracted early variants of the reference Wuhan-Hu-1 strain of SARS-CoV-2 that include mutation D614G (14). Using the same samples, we assessed the degree of concordance between the ELISA results obtained at the UdeM and HQ sites to determine seroconversion. To this effect, we measured the levels of IgG, IgM and IgA specific for the ectodomain of the spike protein as well as the nucleocapsid protein of the SARS-CoV-2 Wuhan-Hu-1 strain.

It has been shown that the affinity of the antibodies produced after an infection with a specific variant of SARS-CoV-2 is affected by mutations present in VoCs (15–17), which differ mostly in the spike ectodomain (18). This information is crucial for public health authorities and for vaccine development, as the extent of cross-reactivity greatly impacts vaccination strategy (19, 20). To verify the suitability of an ‘in-house’ ELISA for informing on the potential for past infection in non-hospitalized individuals to protect against other variants, cross-reactivity ensuing from early infection with SARS-CoV-2 was determined at the UdeM site, against the spike proteins of VoC up to 6 months post-infection. Consistent with this objective, we focused on two important VoCs that emerged in Canada: the P.1 (Gamma) variant, first detected in Brazil in January 2021, and the B.1.617.2 (Delta) variant, first detected in India in December 2020 (21) and dominant in North America and Europe throughout most of 2021 (22, 23).

## 2 Materials and methods

### 2.1 Clinical samples

Clinical samples were obtained from 81 adult volunteers at the Centre Hospitalier Universitaire (CHU) de Québec – Université Laval who provided written informed consent (approved by the “Comité d’éthique de la recherche du CHU de Québec-UL”, registration number 2021-5241). They were selected based on the following criteria: ≥18 years of age; had a PCR-confirmed diagnosis of SARS-CoV-2; had not been admitted to an intensive care unit for COVID-19; and were not hospitalized at enrollment. The participants were stratified by age: 18-49, 50-59, 60-69 and >70 years of age; the age distribution of the study participants is illustrated in **Supplementary Figure 1**. The symptoms reported in each age group were similar (**Supplementary Table 1**). They had 6 visits over a 24-week period: at weeks 3, 4, 8, 12, 16 and 24 post PCR-confirmed diagnosis. A second PCR test was conducted at the time of enrollment and 27 (33%) individuals had a negative test result, suggesting that some had fully recovered while most had not. The results of this second PCR test did not influence eligibility to the current study.

At each visit, 30 mL blood sample was collected in 6 mL tubes (BD Vacutainer 367815). Four participants were lost to follow-up: one aged 18-49 after week 3; one (aged 18-49) after week 8; one (aged 60–69) after week 12; and one (aged 70-79 years) after week 20. One participant did not complete the week 24 visit as she became pregnant.

Enrollment was completed by February 15th, 2021. At that time, early variants of the reference Wuhan-Hu-1 strain of SARS-CoV-2 were still dominant in Québec. During their 24-week sampling window, nine individuals (11%) received the Oxford–AstraZeneca or Pfizer–BioNTech COVID-19 vaccine and provided post-vaccination specimens. All but one were vaccinated between the week 12 and 24 visits; the other individual was vaccinated on weeks 5 and 20.

Negative control sera were collected between June 4th and 9th, and again between July 2nd and 8th 2020 from eight individuals (age range: 20-55 years old, median: 47.5 years; 7 women and 1 man) who had never received a positive test result for SARS-CoV-2. The second round of sampling was made to ensure that no individual had been in early stages of infection during the first round of sampling. No SARS-CoV-2 vaccine was yet available.

The collected blood samples were coded. Sample tubes were gently inverted, held at room temperature for 15–30 min, and spun at 1600 g for 15 min. Serum samples (1 mL aliquots) were transferred into cryovials (Sarstedt Inc., product 72.694.006), frozen in an upright position at -20°C, and stored at -80°C until shipment on dry ice to the assay site, where they were maintained at -80°C up until use.

### 2.2 SARS-CoV-2 viral antigens

The spike protein elicits the strongest humoral response upon infection (24–27) and should be used as a reference in population-based seroprevalence studies (28). Spike protein ectodomains were obtained from the National Research Council of Canada for the following strains: Wuhan-Hu-1 (PRO1-429 (SmT1-1), B.1.617.2 (PRO7176-1 [SmT1(B.1.617.2]), and P.1 (PRO6875-2 [SmT1v3-B]), where they were produced according to protocols reported elsewhere (29–32).

The SARS-CoV-2 nucleocapsid construct from the Wuhan-Hu-1 (GenBank YP_009724397) was C-terminally fused to the tobacco etch virus (TEV) protease-specific cleavage site and to a hexa-His tag by Lemay and coworkers (10).

### 2.3 Enzyme-linked Immunosorbent assays

At the HQ site: The ELISA protocol was based on recently described protocols (11–13). Immulon 2HB 96-well plates (VWR cat. 62402-972 [3455]) were coated with 100 μL of spike antigen (Wuhan-Hu-1 strain) diluted to 2.5 µg/mL in PBS, covered and incubated 16-20 h at 4°C. The plates were washed four times with 300 µL/well of PBS + 0.1% Tween (PBS-T) using an automated 405 TS Microplate Washer (Biotek) according to the manufacturer’s specifications. The plates were blocked with PBS-T + 3% (w/v) milk, kept 60 ± 5 min at room temperature and washed with PBS-T as described above. Clinical serum samples were heat-inactivated for 1 h in a heating block at 55°C, diluted in PBS-T + 1% (w/v) milk (as described in **Table 1**), and added to the plates. After 60 ± 5 min at room temperature, the plates were washed with PBS-T as described above.

**Table 1:** Serum dilutions used in ELISA^a^.

Sample	IgA	IgM	IgG	Total Ig
Clinical serum	1/400	1/200	1/800	1/800
Internal calibrator: HQ UdeM	1/200 1/800	1/200 1/800	1/5 000 1/800	1/10 000 1/800
Negative control: HQ UdeM	1/200 1/400	1/200 1/200	1/200 1/800	1/200 1/800

a Dilutions used unless otherwise specified in the text.

The secondary antibody was diluted in PBS-T + 1% (w/v) milk powder (as described in **Table 2**) and added to the plates, which were kept in the dark for 60 ± 5 min at room temperature and washed with PBS-T as described above. One hundred µL/well of 3,3’,5,5’-tetramethylbenzidine (TMB) (SCY-TM4999, ESBE Scientific) were added, and the plates were incubated 20 ± 1 min in the dark at room temperature. The optical density (OD) was measured at 450 nm (sample) and 630 nm (background) with a Synergy H1 microplate reader (Biotek) within 10 min after adding 1N H_2_SO_4_ to stop the reaction. This corrected OD is hereinafter referred to as ΔOD_450-630_.

**Table 2:** Secondary antibodies used and their dilutions.

Secondary antibody	Study	Dilution
Goat anti-human IgG HRP Life Technologies (Invitrogen) Catalog #31413	HQ; UdeM Fig 1B, 4-6	1/50 000
Goat anti-human IgG (γ-chain-specific) HRP Sigma-Aldrich Catalog #A6029	UdeM Fig 1C	1/10 000
Goat anti-human IgA HRP Jackson ImmunoResearch Catalog #109-036-011	HQ; UdeM Fig 1B, 4-6	1/5 000
Goat Anti-human IgA + IgG + IgM (H+L) HRP (Total Ig) Jackson ImmunoResearch Catalog #109-035-064	HQ; UdeM Fig 1B, 4-6	1/30 000
Goat anti-human IgM (Fc) HRP Jackson ImmunoResearch Catalog #109-035-129	HQ	1/20000
Goat anti-human IgM (µ-chain specific) HRP Sigma-Aldrich Catalog # A6907	UdeM Fig 1B, 4-6	1/10 000

Positive and negative controls were included in triplicate on each plate. A pooled plasma sample was collected from three individuals in 2019 (i.e., before the pandemic) and served as the negative control. An internal standard composed of serum from individuals who tested positive for SARS-CoV-2 (20/162, NISBC) was used as the positive control and to correct for plate-to-plate and day-to-day variations. Samples were diluted in PBS-T + 1% (w/v) milk powder as described in **Table 1**. The results of a plate were excluded when the value of the internal calibrator differed by more than 20% from the mean ΔOD_450-630_ determined from a minimum of 24 OD reads (preparation of three positive controls/operator processed by two different operators). The cut-off value to determine seropositivity was set as the mean ΔOD_450-630_ value (at 1/200 dilution) using 20 negative serum samples plus 3 standard deviations (SD). The cut-off values were 0.408 for total Ig, 0.177 for IgG, 0.314 for IgM, and 0.177 for IgA.

At the UdeM site: The ELISA protocol was based on that described by (12, 13, 33) but included some modifications recently described in (10). The following procedure was applied to all ELISAs performed at UdeM unless otherwise specified. Immulon 1B 96-well plates (Thermo Fisher Scientific) were coated with the antigen of interest as described above for the HQ assay. Specifically, the spike protein from the Wuhan-Hu-1, Delta (B.1.617.2) or Gamma (P.1) variants, and the nucleocapsid protein from the Wuhan-Hu-1 variant were used at the same dilutions as those for the HQ assay. The washing and blocking steps were similar to those performed at the HQ site, except that an automated Biotek 50 TS Microplate Washer was used according to the manufacturer’s specifications. Clinical serum samples were heat-inactivated, diluted, and added as described above for the HQ assay. The serum dilutions are those specified in **Table 1**, except that a 1/50 dilution was used for the longitudinal assessment of IgGs directed against Wuhan-Hu-1 spike.

The secondary antibody was added as at the HQ site; the antibodies and their dilution factors are specified in **Table 2**. Color development used a reagent and inactivation protocol that differed from the HQ site. Here, 100 µL/well of 3,3',5,5'-tetramethylbenzidine (TMB) (T4444, Sigma-Aldrich) were added and the plates were incubated 20 min in the dark at room temperature. The reaction was inactivated by the addition of 2 M HCl, and the OD was immediately measured at 450 nm (signal) and 595 nm (background) with a FLUOstar Optima microplate reader (BMG Labtech); this corrected OD is hereinafter referred to as ΔOD_450-595_. Positive and negative controls were included in triplicate on each ELISA plate.

The internal calibrator consisted in a pool of 13 SARS-CoV-2-positive sera (PCR-confirmed) diluted 1/800 in PBS-T + 3% (w/v) milk powder. The results from a 96-well plate were excluded when the value of the internal calibrator differed by more than 30% from the median value, as indicated below. Under conditions used to longitudinally assess anti-spike (Wuhan-Hu-1) IgG, the median ΔOD_450-595_ of the internal calibrator was 1.24 (± 30% window = 0.87-1.62) based on 44 independent triplicate assays processed by three different operators over 4 months. Under conditions used for isotype assessment in anti-spike assays, the median ΔOD_450-595_ of the internal calibrator was 1.85 (± 30% window = 1.29-2.38) for anti-spike (Wuhan-Hu-1) total Ig and 1.50 (± 30% window = 1.05-1.94) for anti-spike (Wuhan-Hu-1) IgG. For anti-nucleocapsid assays, the median ΔOD_450-595_ of the internal calibrator was 0.25 (± 30% window = 0.18-0.33) for total Ig and 0.45 (± 30% window = 0.32-0.59) for IgG.

The negative controls were sera from eight SARS-CoV-2-negative individuals, which were pooled and diluted in PBS-T + 1% (w/v) milk powder similarly to the SARS-CoV-2-positive samples. The positivity threshold for each dataset was the average ΔOD_450-595_ + 1 × standard deviation (SD) of the negative controls from the relevant assay plates. Specifically, the positivity thresholds were 0.21 for total Ig, 0.14 for IgG, 0.16 for IgM, and 0.15 for IgA.

### 2.4 Statistical analysis

Statistical analysis was done with GraphPad Prism 9™. The normal distribution of the OD data was verified using Bartlett’s tests. All datasets were then compared using a one-way, two-sided, non-parametric Kruskal-Wallis ANOVA test. Where differences between datasets were significant, the datasets were then compared using a two-sided *post-hoc* Dunn’s test to identify the groups that differ significantly from the others. Receiver operating characteristic (ROC) analysis was performed using Graphpad Prism 9.0.0.

## 3 Results and discussion

Volunteers were recruited in the Québec City region (Canada) before February 15th, 2021. At that time, the Alpha and Beta variants of SARS-CoV-2 were in circulation but the early variants of the Wuhan-Hu-1 strain of SARS-CoV-2 remained dominant (14). Although estimates vary widely by regions and populations, early variants of the Wuhan-Hu-1 strain, generally characterized by the presence of the D614G and a few additional mutations, produced mild to moderate illness and approximately 5% of patients experienced severe symptoms necessitating intensive care (34–40). The Delta (B.1.617.2) and Gamma (P.1) strain of SARS-CoV-2, investigated here, were not yet in circulation.

Serum samples were collected from 81 non-hospitalized, SARS-CoV-2-positive (PCR-confirmed) individuals in four age groups: 18-49 (n = 33); 50-59, 60-69, and 70+ (n = 16 each), at weeks 3, 4, 8, 12, 16 and 24 post-diagnosis. They had a positive PCR test result for SARS-CoV-2 ∼3 weeks (average: 17.25 days) prior to enrollment. One participant was asymptomatic. All other volunteers were mildly symptomatic, with an average of 3 symptoms among fever, myalgia, headache, sore throat, newly developed smell or taste disorder, cough, or difficulty breathing. Participants generally remained engaged, most (94%) attending all visits; 2 individuals attended only 1 or 2 visits and were excluded from analysis. Eight individuals had received one dose of a SARS-CoV-2 vaccine by week 24, one had received a single dose by week 8 and two doses by week 24. The results of post-vaccination visits were treated separately.

Shortly after the outset of the first wave of SARS-CoV-2 in Canada, in-house ELISA protocols were developed at the HQ and UdeM sites. The HQ site has experience with blood tests and ELISAs and adapted their protocol to SARS-CoV-2 antigens. The UdeM site had limited experience with antibody detection by ELISA, such that this study compared laboratories having different levels of initial preparedness. The protocols were generally similar but differed as follows: in the type of 96-well microtiter plate, the model of automated plate-washer, the spectrophotometric plate-reading device, the source and dilution of some secondary antibodies, the composition of the TMB colorimetric reagent and reaction quenching procedure, and (in one protocol) the serum dilution. Using these protocols, total Ig levels that targeted Wuhan-Hu-1 spike protein were measured, as were the levels of IgG, IgA and IgM.

### 3.1 Longitudinal assessment of anti-spike IgG at the HQ and UdeM sites

Spike-specific IgGs were longitudinally assessed to study their dynamics in non-hospitalized individuals. The results were also compared between the two study sites, which used different ELISA protocols. A subset of 50 individuals was selected to balance the age distribution of the 70 unvaccinated individuals who provided samples regularly.

**Figure 1:**
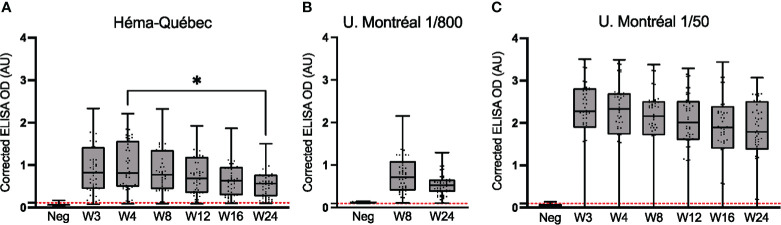
Comparative longitudinal assessment of anti-spike (Wuhan-Hu-1 strain) IgG. ELISAs using serum samples from n = 50 SARS-CoV-2-positive (PCR-confirmed) individuals who remained unvaccinated throughout the study and attended all or most visits. **(A)** Assays were performed for samples collected from weeks 3 to 24 at the HQ site. Serum dilutions used in **(A)** are listed in **Table 1**. **(B)** The subset of assays for weeks 8 and 24 was repeated at the UdeM site, using the same clinical samples, dilution and secondary antibody as in **(A)**. Serum was at 1/800 dilution (**Table 1**). **(C)** Assays for weeks 3 to 24 were repeated at the UdeM site, using a different secondary antibody and different dilution of the antibody, and a 1/50 dilution of serum. Anti-IgG secondary antibodies used are listed in **Table 2**. The average of triplicate assays is given as corrected OD. The median is shown in the quartile boxplots where the whiskers include all values (no outliers excluded). n: Negative controls. Positivity threshold (red dashed line): HQ = 0.113; UdeM = 0.097. *p < 0.05.

At the HQ site, seroconversion was observed for 49 of the 50 individuals (98.0%) at the week 4 visit; this result was confirmed at all time points tested (**Figure 1A**). The median serum anti-spike IgG signal was highest at week 4 and decreased thereafter (p < 0.05). The observed signal at week 24 demonstrates that serum anti-spike IgG persisted at least 6 months post-infection in this cohort.

These results were successfully reproduced by at the UdeM site, who repeated the week 8 and 24 experiments. The same clinical samples, dilution and secondary antibody were used here and gave essentially indistinguishable results although instruments, color development and other protocol details were unique to each test site. (**Figures 1A** vs. **B**). The individual identified as seronegative at the HQ site was also identified as such in this repeat experiment; moreover, one more individual was seronegative at week 8 but not week 24. This excellent concordance validates the reproducibility of the assay. ROC analyses were performed using the OD values obtained at each test site, for each visit (**Supplementary Table 2**).

To verify experimental agreement when different materials and conditions are used, the same experiment was performed using a different source and dilution of secondary antibody (**Table 2**), different materials for color development (see Methods), and a different serum dilution (i.e., a 1/50 dilution was used instead of the 1/800 dilution; **Figure 1C**). The difference in assay conditions, particularly serum dilution, is evident, with the corrected absorbance ∼2.5-fold greater (**Figures 1A, B** vs. **C**).

Despite this difference in signal intensity (**Figures 1A** vs. **C**), the IgG titers trended similarly, peaking at week 4 and slowly decreasing thereafter; again, the signal at week 24 confirms the persistence of serum anti-spike IgG over at least 6 months, as observed in population-level studies of symptomatic and asymptomatic SARS-CoV-2-positive cohorts (41, 42). Importantly, the seroconversion sensitivity of both ELISA protocols was nearly identical (**Supplementary Table 2**: HQ = 97% *vs* UdeM = 96%): among the 50 individuals tested, the previously identified individual was confirmed as seronegative at all weeks in both assays, and one additional individual was identified as seronegative at all weeks (**Figure 1C**). These results, obtained during a public health crisis, validate that seroconversion can be reliably determined at independent test sites, and with different reagents, reagent sources, instrumentation, and sample or reagent dilutions, for decentralized population-level immune surveillance.

### 3.2 Impact of age and sex on the production of anti-spike IgG

**Figure 2:**
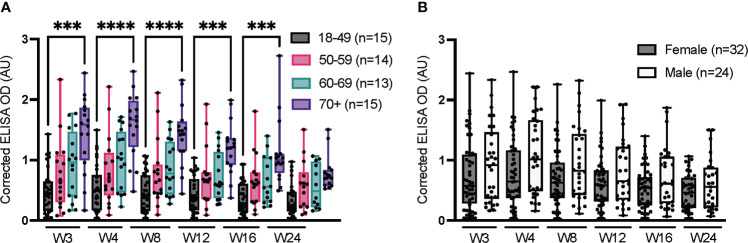
Impact of age and sex on serum anti-spike IgG over 24 weeks. ELISAs were conducted at the HQ site using serum samples from confirmed SARS-CoV-2-positive individuals, as in **Figure 1**. **(A)** Stratification according to age. **(B)** Stratification according to sex; the sex of one individual is unknown and was not considered in this analysis. The average of triplicate assays is given as corrected OD. The median is shown in the quartile boxplots where the whiskers include all values (no outliers excluded). ***p < 0.001; ****p < 0.0001.

The results of the longitudinal, anti-spike IgG ELISAs reported in **Figure 1A** (data from the HQ site) were stratified according to age and sex (**Figure 2**). Consistent with prior reports (10, 43, 44), the median IgG response to infection with SARS-CoV-2 tended to increase with age (**Figure 2A**). The signal for IgG was significantly higher for the oldest participants (≥70 years) than the youngest (18-49 years) from weeks 3 through 16 (*i.e*., statistical significance was lost at week 24). This moderate but positive correlation between IgG level and age in adults in response to infection with SARS-CoV-2 is consistent with other reports (43, 44). This age-related correlation with IgG level contrasts with the effects of vaccine-related immunity, where the IgG response is lower in the elderly population relative to older adults. That effect is particularly marked in the over-80 age group, which we did not specifically investigate, reportedly due to immunosenescence and inflammaging (chronic inflammation that develops with advanced age) that reduce antibody production following vaccination (45, 46). The median IgG response was slightly higher in men than women, although this difference was not statistically significant, as reported in other studies (**Figure 2B**) (47–49).

### 3.3 Vaccination following infection strongly increases humoral immunity in non-hospitalized individuals

Throughout the study, eight individuals received one dose of a SARS-CoV-2 vaccine between weeks 12 and 24 after a positive PCR test result, and one received two doses on weeks 5 and 20 after a positive test. Post-infection IgG levels had waned prior to vaccination in all but one individual (**Supplementary Figure 2**).

A first dose of vaccine clearly boosted IgG levels in these previously infected, non-hospitalized individuals (**Supplementary Figure 2**). Although a single individual received two vaccine doses, the benefit of a second dose was clear as it boosted IgG levels following some waning of the first dose. The median IgG signal resulting from this second (or third, in one case) antigen response to SARS-CoV-2 (median ELISA OD = 2.3) was even greater than that of the 70+ year-old group at week 4 (**Figure 2**, median ELISA OD = 1.67), despite the younger age of the infected then vaccinated individuals (average 54 years old). Although few individuals were studied, these results confirm the beneficial impact of vaccination on the humoral response, as reported in other studies (50–52).

### 3.4 Longitudinal assessment of anti-spike total Ig, and IgM and IgA

**Figure 3:**
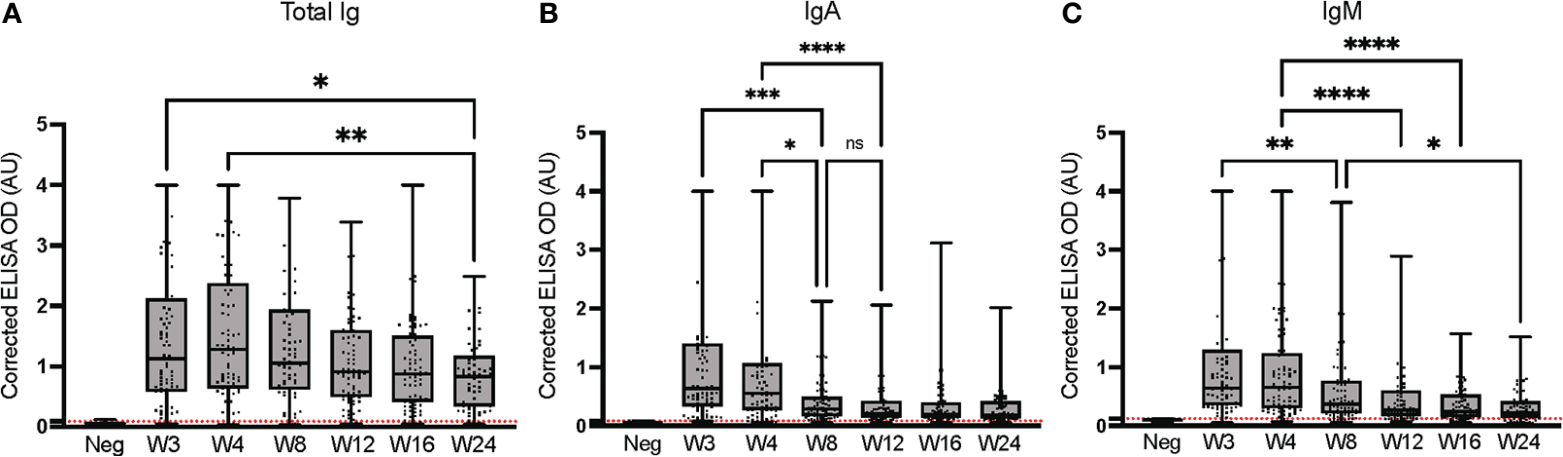
Longitudinal assessment of anti-spike (Wuhan-Hu-1 strain) immunoglobulins at the HQ site. ELISAs were conducted at the HQ site using serum samples from 79 PCR-confirmed SARS-CoV-2-positive individuals. **(A)** Total Ig; **(B)** IgA; **(C)** IgM. The post-vaccination samples of the nine individuals who were vaccinated during the study were excluded. The secondary antibodies are listed in **Table 2**. The average of triplicate assays is given as a corrected OD. The average of triplicate assays is given as corrected OD. The median is shown in the quartile boxplots where the whiskers include all values (no outliers excluded). Negative controls using serum from eight SARS-CoV-2-negative individuals are given as the average of triplicate assays. ns: not significant; *p < 0.05; **p < 0.01; ***p < 0.001; ****p < 0.0001. Neg: Negative controls. Positivity threshold (red dashed line) for total Ig: 0.091; IgA: 0.083; IgM: 0.123. Results for the seroconversion ROC analyses for these datasets are presented in **Supplementary Table 3**.

We longitudinally assessed anti-spike (Wuhan-Hu-1) total Ig, IgA and IgM over 24 weeks to examine dynamics of antibody titer in SARS-CoV-2-positive (PCR-confirmed), non-hospitalized individuals (**Figure 3**). As previously observed (53), the total Ig response to SARS-CoV-2 was predominantly accounted for by IgG (**Figure 1**), with a weaker contribution of IgA and IgM (54–56). The response peaked at week 4 for total Ig, at week 3 for IgA, and was similar at weeks 3 and 4 for IgM. Regardless of the isotype considered, the signal significantly decreased between weeks 3-4 and week 24; the IgA signal waned most rapidly, reaching scarcely detectable levels in the majority of individuals by week 12. ROC analysis results for seroconversion are presented in **Supplementary Table 3**. Our observations are consistent with the more rapid production and waning of IgM and IgA than total Ig and IgG upon infection by SARS-CoV-2, as observed in other studies (53–57).

Results of seroconversion were compared at an early (week 8) and a late (week 24) time point for the two test sites (**Table 3**). At week 8, total Ig identified 91% of positive samples at the HQ site, and 89% at the UdeM site; at week 24, both test sites identified 91% of positive samples. For IgG, 94% of the samples tested positive at weeks 8 and 24 at the HQ site, compared to 94% at week 8 and 96% at week 24 at the UdeM site. We note that three among the four samples that tested negative at the HQ site on weeks 8 and 24 also tested negative at the UdeM site. This demonstrates the excellent consistency of both protocols to detect anti-SARS-CoV-2 antibodies.

**Table 3:** Seroconversion in SARS-COV-2-positive individuals determined on weeks 8 and 24 at each test site, according to total Ig, IgG, IgA or IgM anti-spike (Wuhan-Hu-1) response.

		HQ		UdeM	
		n	(%)	n	(%)
Total Ig	Week 8 (N=65)	59	(91%)	58	(89%)
	Week 24 (N=67)	61	(91%)	61	(91%)
IgG	Week 8 (N=65)	61	(94%)	61	(94%)
	Week 24 (N=67)	63	(94%)	64	(96%)
IgA	Week 8 (N=65)	57	(88%)	53	(82%)
	Week 24 (N=67)	55	(82%)	67	(100,0%)
IgM	Week 8 (N=65)	47	(72%)	55	(85%)
	Week 24 (N=67)	32	(48%)	61	(91%)

IgA seropositivity also concorded well at week 8 (HQ: 87.7%, UdeM: 81.5%), but differed somewhat at week 24 (HQ: 82%, UdeM: 100%) (**Table 3**). IgM positivity differed modestly at week 8 (HQ: 72.3%, UdeM: 84.6%) and more significantly at week 24 (HQ: 47.7%, UdeM: 91%); **Table 3**). This is consistent with the results of IgG or total Ig being more reliable to determine seroconversion, as is accepted practice (53–58). Indeed, at week 24 the signals for IgA and IgM are so weak as to cluster near the positivity threshold where their assignment as positive or negative was not so robust as for the remainder of the datasets. This is reflected in the ROC analyses; in general, the sensitivity and specificity of the UdeM and HQ assays for isotyping against the Wuhan-Hu-1 spike antigen were highly consistent (**Supplementary Tables 2**–**4**).

### 3.5 Isotyping of anti-spike and anti-nucleocapsid immunoglobulins

**Figure 4:**
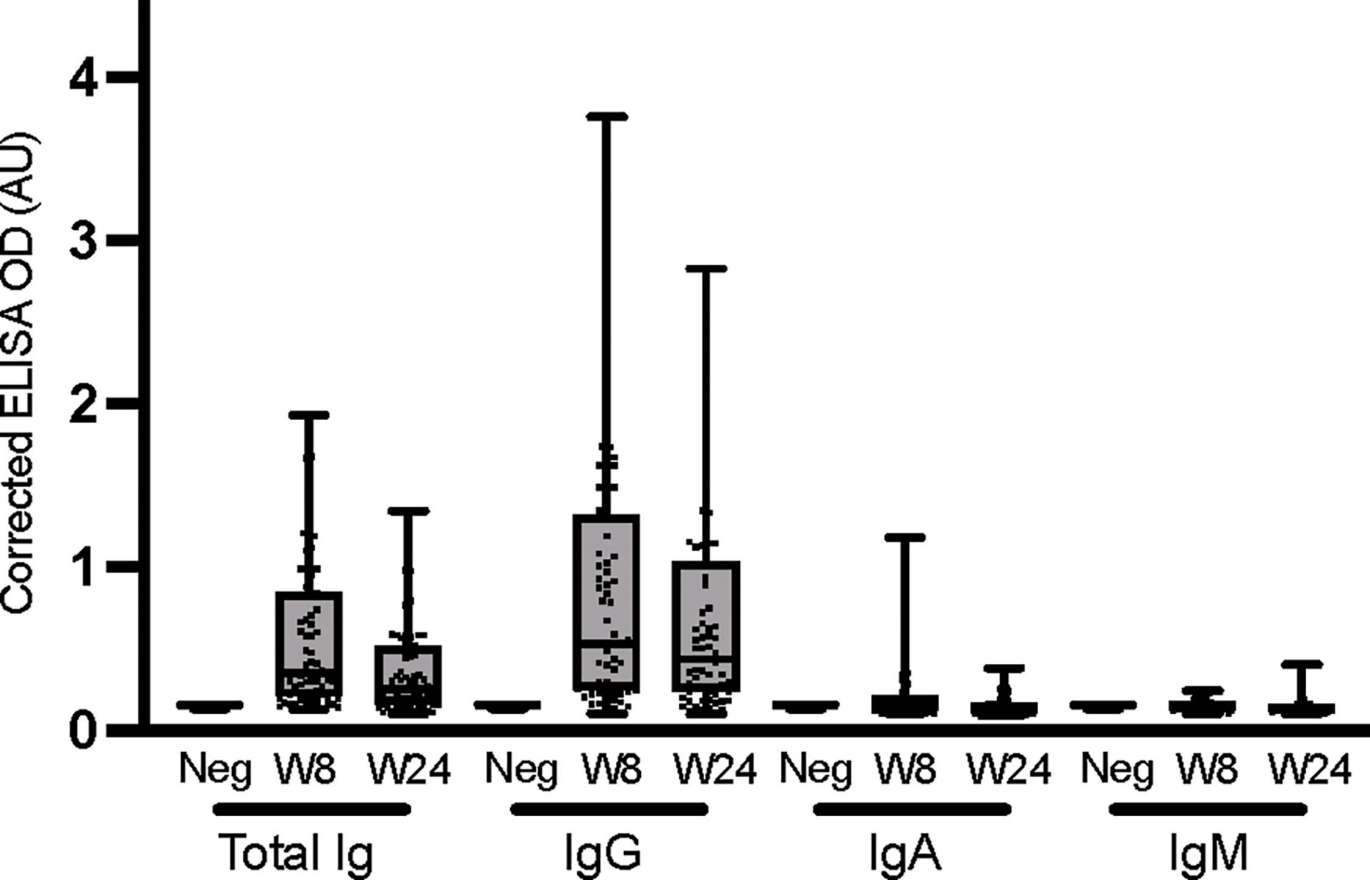
Isotyping of anti-nucleocapsid immunoglobulins. ELISAs were conducted at the UdeM site to determine total Ig, IgG, IgA and IgM against the nucleocapsid (NC) antigen at weeks 8 and 24. Serum samples exclude those of individuals who were vaccinated during the study and include W8: n = 65 and W24: n = 67 individuals. The secondary antibodies used are listed in **Table 2**. The average of triplicate assays is given as corrected OD. The median is shown in the quartile boxplots where the whiskers include all values (no outliers excluded). Negative controls using serum from 8 COVID-negative individuals are given as the average of triplicate assays. Results for the seroconversion ROC analyses for these datasets are presented in **Supplementary Table 5**.

The anti-nucleocapsid response was also determined at the UdeM site for the different immunoglobulins, at an early and a late time point (**Figure 4**). The levels of nucleocapsid-specific total Ig and IgG appeared to decrease over time (although the trend was not statistically significant), and IgA and IgM were undetectable, consistent with prior studies (59–61). Taken together, these data show that the anti-spike response was dominant and persistent following infection with SARS-CoV-2 in this cohort, and provided a more reliable picture of the humoral immune response than did the anti-nucleocapsid response. All immunoglobulin types specific to spike antigen were produced at varying levels and persistence post-infection. By contrast, the anti-nucleocapsid response was weaker and was dominated by IgGs as reported by Sun et al. (62). ROC analyzes for seroconversion were carried out for the isotyping performed against the nucleocapsid antigen at week 8 and 24. The sensitivity and specificity performance of the ROCs are shown in **Supplementary Table 5**. Assays that varied in their location, operators and/or materials yielded highly concordant results, which validates the relevance of performing decentralized immune surveillance in non-hospitalized individuals.

### 3.6 Cross-reactivity with the spike proteins from the Delta and Gamma variants

We next investigated how the immunoglobulins ensuing from an early infection with SARS-CoV-2 in non-hospitalized individuals may cross-react with the spike proteins of VoC. ELISAs were performed at the UdeM site against the spike protein of the Wuhan-Hu-1, Delta (B.1.617.2), and Gamma (P.1) strains, using samples collected at weeks 8 and 24 to assess the persistence of cross-reactivity.

**Figure 5:**
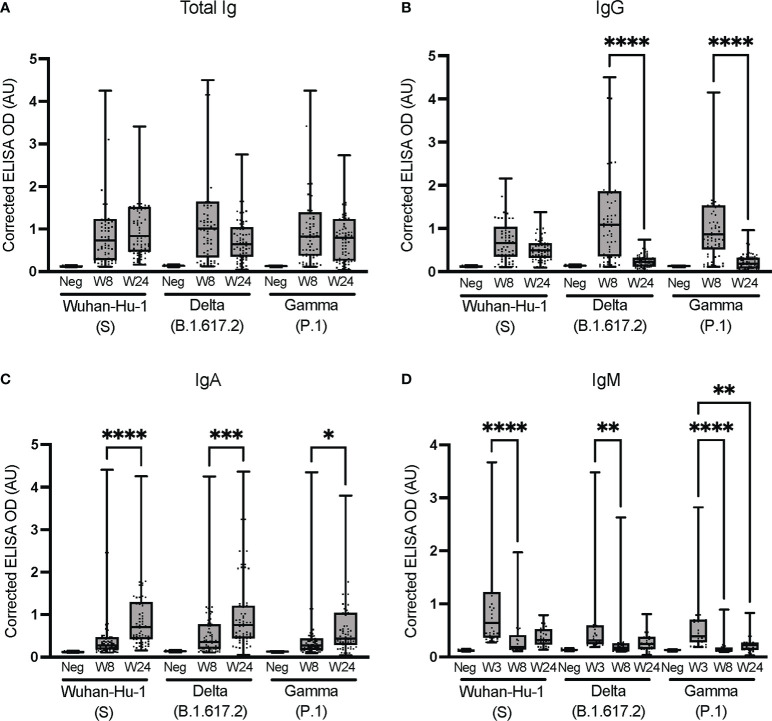
Cross-reactivity against the spike proteins of Wuhan-Hu-1 (S) and the Delta (B.1.617.2) and Gamma (P.1) VoC. Isotyping was conducted at the UdeM site to determine **(A)** Total Ig, **(B)** IgG, **(C)** IgA and **(D)** IgM against the spike protein of VoC. Serum samples are from the same individuals as in **Figure 3** and include W8: n = 65 and W24: n = 67 individuals except for B.1.617.2 IgA at W24 where n = 64. For IgM, 30 samples were tested at W3, W8 and W24. The secondary antibodies are listed in **Table 2**. The average of triplicate assays is given as ΔOD_450-595_ (corrected OD). The average of triplicate assays is given as corrected OD. The median is shown in the quartile boxplots where the whiskers include all values (no outliers excluded). Negative controls (Neg) using serum from eight SARS-CoV-2-negative individuals are given as the average of triplicate assays. *p < 0.05; **p < 0.01; ***p < 0.001; ****p < 0.0001. Results for the seroconversion ROC analyses for these datasets are presented in **Supplementary Table 6**.

The antibodies elicited infected individuals strongly cross-reacted with the Delta (B.1.617.2) and Gamma (P.1) spike proteins (**Figure 5**), consistent with other reports (63). Delta- and Gamma-specific IgGs waned significantly faster (p < 0.0001) than Wuhan-Hu-1-specific IgGs, both in terms of median signal (**Figure 5B**) and number of individuals giving rise to a positive signal (**Supplementary Table 7**). The cross-reactive signals for IgA and IgM were weaker that total Ig and IgG (**Figures 5C**, **D**). Although the Delta- and Gamma-specific IgA signals exhibited a statistically significant increase, the signals were weak and should thus be cautiously interpreted. Despite their low median signals (**Figure 5C**), IgAs specific to at least one of the Delta or Gamma VoC allowed for most SARS-CoV-2-positive individuals to be identified. In contrast, the low signals obtained for IgM at weeks 8 and 24 were unreliable to identify seropositive individuals, consistent with other reports of rapidly waning IgM titers (54, 60). However, the data acquired for IgM at an earlier time point (week 3; **Figure 5C**) allowed for the determination of seropositivity, regardless of the antigen used (**Supplementary Table 7**). This illustrates that IgM levels measured by ELISA cannot identify individuals who were infected more than 3 weeks prior. Seroconversion ROC analyses for cross-reactivity against the spike proteins of Wuhan-Hu-1(S), Delta (B.1.617.2) and Gamma (P.1) VoC confirmed that the assays offered good sensitivity and specificity for the detection of total Ig and IgG early after the infection, independently of the VoC antigen considered (**Supplementary Table 5**). The assay performances tended to decrease over time and be more variable for IgA and IgM, consistent with their weaker signals.

These data demonstrate that all antibody isotypes were elicited as part of the anti-SARS-CoV-2 immune response of non-hospitalized individuals with an early warning of IgM, consistent with other reports (33, 64). The antibodies elicited in individuals infected with early variants of the reference Wuhan-Hu-1 strain of SARS-CoV-2 strongly cross-reacted with the Delta (B.1.617.2) and Gamma (P.1) spike proteins soon after infection, but less so after 6 months. Along with the post-vaccination results (**Supplementary Figure 2**), this is consistent with regular vaccine boosters being required to sustain the humoral response in the face of new VoC (65, 66).

**Figure 6:**
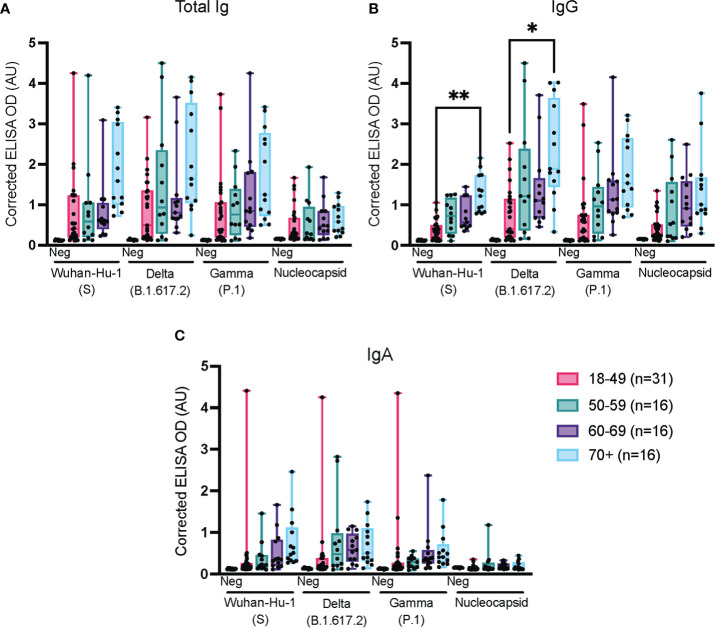
Impact of age on the immune response against spike and nucleocapsid antigens. ELISAs for total Ig, IgG and IgA were conducted at the UdeM site using the serum samples from **Figure 3**, week 8. The 65 confirmed SARS-CoV-2-positive individuals were grouped as: 18-49 years of age (n=27); 50-59 (n=12); 60-69 (n=13) and 70+ years of age (n=13). Post-vaccination samples were excluded. The secondary antibodies used are listed in **Table 2**. The average of triplicate assays is given as ΔOD_450-595_ (corrected OD). The average of triplicate assays is given as corrected OD. The median is shown in the quartile boxplots where the whiskers include all values (no outliers excluded). Negative controls (Neg) using serum from 8 COVID-negative individuals are shown as the average of triplicate assays. *p < 0.05 ** p < 0.01.

We further stratified the above data obtained at week 8 post-infection according to age (**Figure 6**); as previously mentioned, the results of the IgM analysis at week 8 were excluded because the signal was too low to be interpreted. For total Ig, IgG and IgA, the median antibody levels tended to increase with age, as did the cross-reactivity with the Delta (B.1.617.2) and Gamma (P.1) VoC (**Figure 5**). At variance with the results obtained with the HQ assay protocol for a subset of these samples (**Figure 2**), the increase was rarely statistically significant when assayed with the UdeM assay protocol (**Figure 5**). Nonetheless, the same trend was observed using both ELISA protocols, further confirming that different protocols can successfully report on various aspects of the humoral response to viral infection in a non-hospitalized population.

## 4 Conclusions

In this study, two anti-SARS-CoV-2 ELISAs protocols and modifications thereof yielded highly concordant seroconversion results even though they were developed independently and established at decentralized test sites in different cities and performed using different reagents and instrumentation. The observed cross-reactivity of antibodies elicited in individuals infected with early variants of the reference Wuhan-Hu-1 strain of SARS-CoV-2 against VoCs and the post-vaccination humoral response are in agreement with the current literature, indicating robustness of the assays. This study confirms that robust immune surveillance can be rapidly and reliably established in urgent situations when centralized testing is not available. Furthermore, the study cohort was representative of the majority of the population of Canada where approximately 95% of infected adults were not hospitalized (6) indicating that results from such studies may be a valuable asset in informing public health decision-makers.

## Data availability statement

Publicly available datasets were analyzed in this study. This data can be found here: https://www.ncbi.nlm.nih.gov/protein/YP_009724397.

## Ethics statement

The studies involving human participants were reviewed and approved by Comité d’éthique de la recherche du CHU de Québec-UL (registration number 2021-5241). The patients/participants provided their written informed consent to participate in this study.

## Author contributions

AD, EL, M-FP, M-PC, and FD performed all experiments; AD, EL, M-FP, M-PC, MdG, DBr, and JP performed data analysis; EL performed statistical analysis; MS, CG, and YD produced antigens; ST coordinated sample collection; ST, DBo, JFM, DB, and JP designed the study; M-FP, M-PC, and JP led the literature review; M-FP and JP drafted the manuscript, aided by all authors. All authors revised and approved the manuscript.
