# Supplementary material for: Assessment of the longitudinal humoral response in non-hospitalized SARS-CoV-2-positive individuals at decentralized sites: Outcomes and concordance
Source: Front Immunol. 2023 Jan 20;13:1052424. doi: 10.3389/fimmu.2022.1052424 (PMC9895839; doi:10.3389/fimmu.2022.1052424)
Supplement: Supplementary file 1 [file DataSheet_1.pdf]

Supplementary Material

Assessment of the longitudinal humoral response in non-hospitalized SARS-CoV-2-positive individuals at decentralized sites: Outcomes and concordance

Abdelhadi Djaïleb, Étienne Lavallée, Megan-Faye Parker, Marie-Pierre Cayer, Florence Desautels, Marie Joëlle de Grandmont, Matthew Stuible, Christian Gervais, Yves Durocher, Sylvie Trottier, Denis Boudreau, Jean-François Masson, Danny Brouard, and Joelle N. Pelletier

Table S1: Reported symptoms in 80 non-hospitalized, SARS-CoV-2-positive (PCR-confirmed) adults exhibiting mild to moderate COVID-19 symptoms whose serological assessment was performed.

AGE		Fever	Cough	Respiratory difficulties	Loss of smell	Loss of taste	Muscle pain	Sore throat	Fatigue	Runny nose/ congestion	Headache	Diarrhea
**18-49**	(n = 32)	8	16	10	19	16	11	8	20	11	11	6
**50-59**	(n = 16)	8	7	8	9	8	8	6	8	6	11	10
**69-69**	(n = 16)	6	9	6	6	6	5	0	11	4	10	4
**70+**	(n = 16)	8	10	5	5	8	2	3	14	5	5	7
	Total:	30	42	29	39	38	26	17	53	26	37	27

Table S2: Receiver operating characteristic (ROC) analysis of comparative longitudinal assessment of anti-spike (Wuhan-Hu-1 strain) IgG (Figure 1).

Fig 1A

HQ site	Threshold[a]	Sensitivity	95% CI	Specificity	95% CI
W3	> 0.129	96	87% to 99%	86	49% to 99%
W4	> 0.115	98	89% to >99%	86	49% to 99%
W8	> 0.112	98	90% to >99%	86	49% to 99%
W12	> 0.128	96	87% to 99%	86	49% to 99%
W16	> 0.130	96	87% to 99%	86	49% to 99%
W24	> 0.114	98	90% to >99%	86	49% to 99%

Fig 1B

UdeM 1/800	Threshold [a]	Sensitivity	95% CI	Specificity	95% CI
W8	> 0.144	94	84% to 98%	82	52% to 97%
W24	> 0.144	96	87% to 99%	82	52% to 97%

Fig 1C

UdeM 1/50	Threshold[a]	Sensitivity	95% CI	Specificity	95% CI
W3	> 0.118	96	87% to 99%	88	53% to 99%
W4	> 0.118	96	86% to 99%	88	53% to 99%
W8	> 0.118	96	87% to 99%	88	53% to 99%
W12	> 0.118	96	87% to 99%	88	53% to 99%
W16	> 0.118	96	87% to 99%	88	53% to 99%
W24	> 0.113	96	87% to 99%	88	53% to 99%

[a] Threshold: the threshold value was selected to be the nearest approximation to the positivity threshold determined for each dataset. Threshold for Fig1A = 0.113; Fig1B = 0.144; Fig1C = 0.097.

Table S3: Receiver operating characteristic (ROC) analysis for longitudinal assessment of anti-spike (Wuhan-Hu-1 strain) immunoglobulins at the HQ site (Figure 3).

Total Ig	Threshold [a]	Sensitivity	95% CI	Specificity	95% CI
W3	> 0.100	96	89% to 99%	88	53% to 99%
W4	> 0.0990	96	89% to 99%	88	53% to 99%
W8	> 0.0960	96	89% to 99%	88	53% to 99%
W12	> 0.0935	96	89% to 99%	88	53% to 99%
W16	> 0.112	95	87% to 98%	88	53% to 99%
W24	> 0.0940	96	88% to 99%	88	53% to 99%

IgA	Threshold [a]	Sensitivity	95% CI	Specificity	95% CI
W3	> 0.0875	99	93% to 99%	>99	68% to >99%
W4	> 0.0830	96	89% to 99%	>99	68% to >99%
W8	> 0.0855	94	86% to 97%	>99	68% to >99%
W12	> 0.0850	92	84% to 96%	>99	68% to >99%
W16	> 0.0855	93	86% to 97%	>99	68% to >99%
W24	> 0.0840	94	86% to 98%	>99	68% to >99%

IgM	Threshold [a]	Sensitivity	95% CI	Specificity	95% CI
W3	> 0.123	98	91% to 99%	88	53% to 99%
W4	> 0.123	97	91% to 99%	88	53% to 99%
W8	> 0.123	90	81% to 95%	88	53% to 99%
W12	> 0.123	85	75% to 91%	88	53% to 99%
W16	> 0.123	86	76% to 92%	88	53% to 99%
W24	> 0.123	79	68% to 87%	88	53% to 99%

[a] Threshold: the threshold value was selected to be the nearest approximation to the positivity threshold determined for each dataset. Thresholds for Fig 3: Total Ig = 0.091; IgA = 0.083; IgM = 0.123.

Table S4: Receiver operating characteristic (ROC) analysis for isotyping of anti-spike (Wuhan-Hu-1 strain) immunoglobulins at the UdeM site (Table 3).

Spike		Threshold [a]	Sensitivity	95% CI	Specificity	95% CI
**Total Ig**	W8	> 0.212	85	76% to 92%	91	62% to 99%
	W24	> 0.212	94	86% to 97%	91	62% to 99%
**IgG**	W8	> 0.144	91	81% to 96%	73	43% to 90%
	W24	> 0.144	93	84% to 97%	73	43% to 90%
**IgA**	W8	> 0.155	84	74% to 91%	82	52% to 97%
	W24	> 0.158	99	93% to >99%	82	52% to 97%
**IgM**	W8	> 0.165	46	35% to 58%	91	62% to >99%
	W24	> 0.165	88	78% to 94%	91	62% to >99%

[a] Threshold: the threshold value was selected to be the nearest approximation to the positivity threshold determined for each dataset. Thresholds for UdeM data in Table 3: Total Ig = 0.212; IgG = 0.144; IgA = 0.154; IgM = 0.162.

Table S5: Receiver operating characteristic (ROC) analysis for isotyping of anti-nucleocapsid immunoglobulins at the UdeM site (Figure 4).

Nucleocapsid		Threshold [a]	Sensitivity	95% CI	Specificity	95% CI
**Total Ig**	W8	> 0.127	>99	95% to >99%	88	53% to 99%
	W24	> 0.127	87	78% to 93%	88	53% to 99%
**IgG**	W8	> 0.171	90	80% to 95%	>99	68% to >99%
	W24	> 0.168	90	81% to 95%	>99	68% to >99%
**IgA**	W8	> 0.105	99	92% to >99%	75	41% to 97%
	W24	> 0.104	90	81% to 95%	75	41% to 97%
**IgM**	W8	> 0.128	60	42% to 75%	75	41% to 96%
	W24	> 0.130	43	27% to 61%	75	41% to 96%

[a] Threshold: the threshold value was selected to be the nearest approximation to the positivity threshold determined for each dataset. Thresholds for Fig 4 : Total Ig = 0.123; IgG = 0.168; IgA = 0.104; IgM = 0.128.

Table S6: Receiver operating characteristic (ROC) analysis for cross-reactivity against the spike proteins of Wuhan-Hu-1 (S) and the Delta (B.1.617.2) and Gamma (P.1) VoC (Figure 5).

Total Ig		Threshold	Sensitivity	95% CI	Specificity	95% CI
Wuhan-Hu-1	W8	> 0.212	84	74% to 91%	88	53% to 99%
	W24	> 0.212	93	85% to 97%	88	53% to 99%
Delta	W8	> 0.146	93	84% to 97%	88	53% to 99%
	W24	> 0.144	90	81% to 95%	88	53% to 99%
Gamma	W8	> 0.113	>99	95% to >99%	88	53% to 99%
	W24	> 0.113	92	83% to 96%	88	53% to 99%

IgG		Threshold	Sensitivity	95% CI	Specificity	95% CI
Wuhan-Hu-1	W8	> 0.144	92	82% to 96%	75	41% to 96%
	W24	> 0.144	93	84% to 97%	75	41% to 96%
Delta	W8	> 0.156	94	85% to 98%	88	53% to 99%
	W24	> 0.152	69	57% to 79%	88	53% to 99%
Gamma	W8	> 0.144	93	84% to 97%	88	53% to 99%
	W24	> 0.150	58	46% to 69%	>99	67% to >99%

IgA		Threshold	Sensitivity	95% CI	Specificity	95% CI
Wuhan-Hu-1	W8	> 0.155	82	712% to 90%	75	41% to 96%
	W24	> 0.158	97	92% to >99%	75	41% to 96%
Delta	W8	> 0.140	87	76% to 93%	>99	68% to >99%
	W24	> 0.152	96	88% to 99%	>99	68% to >99%
Gamma	W8	> 0.124	90	80% to 95%	75	41% to 96%
	W24	> 0.127	93	84% to 97%	88	53% to 99%

IgM		Threshold	Sensitivity	95% CI	Specificity	95% CI
Wuhan-Hu-1	W3	> 0.165	>99	89% to >99%	88	53% to 99%
	W8	> 0.164	57	39% to 73%	88	53% to 99%
	W24	> 0.165	93	79% to 99%	88	53% to 99%
Delta	W3	> 0.132	>99	89% to >99%	88	53% to 99%
	W8	> 0.132	67	49% to 81%	88	53% to 99%
	W24	> 0.132	88	70% to 95%	88	53% to 99%
Gamma	W3	> 0.121	>99	89% to >99%	88	53% to 99%
	W8	> 0.120	60	42% to 75%	88	53% to 99%
	W24	> 0.122	83	66% to 93%	88	53% to 99%

[a] Threshold: the threshold value was selected to be the nearest approximation to the positivity threshold determined for each dataset. Thresholds for Fig 5: Total Ig (Wuhan-Hu-1 = 0.212; Delta = 0.143; Gamma = 0.110); IgG (Wuhan-Hu-1 = 0.144; Delta = 0.149; Gamma = 0.142); IgA (Wuhan-Hu-1 = 0.154; Delta = 0.136; Gamma = 0.124); IgM (Wuhan-Hu-1 = 0.162; Delta = 0.131; Gamma = 0.119).

Table S7. Seroprevalence rates of total Ig, IgG, IgA and IgM against spike protein of Wuhan-Hu-1, B.1.617.2 and P.1 variants of SARS-CoV-2, as determined by ELISA.

SARS-CoV-2 variant:		Wuhan-Hu-1			Delta (B.1.617.2)			Gamma (P.1)		
Isotype	Weeks post-infection	3	8	24	3	8	24	3	8	24
Total Ig [a]	Positive samples	/	92.3% (n=60)	100% (n=67)	/	92.3% (n=62)	89.5% (n=60)	/	93.9% (n=61)	89.5% (n=60)
	Negative samples	/	7.7% (n=5)	0% (n=0)	/	7.7% (n=5)	10.5% (n=7)	/	6.1% (n=4)	10.5% (n=7)
IgG [a]	Positive samples	/	90.8% (n=59)	92.6% (n=62)	/	95.4% (n=62)	68.7% (n=46)	/	93.9% (n=61)	56.7% (n=38)
	Negative samples	/	9.2% (n=6)	7.4% (n=5)	/	4.6% (n=3)	31.3% (n=21)	/	6.1% (n=4)	43.3% (n=29)
IgA [b]	Positive samples	/	84.6% (n=55)	100% (n=64)	/	84.6% (n=55)	95.3% (n=61)	/	84.6% (n=55)	92.2% (n=59)
	Negative samples	/	15.4% (n=10)	0% (n=0)	/	15.4% (n=10)	4.7% (n=3)	/	15.4% (n=10)	7.8% (n=5)
IgM [c]	Positive samples	100% (n=30)	76.7% (n=23)	96.7% (n=29)	100% (n=30)	56.7% (n=17)	73.3% (n=22)	100% (n=30)	43.3% (n=13)	70% (n=21)
	Negative samples	0% (n=0)	23.3% (n=7)	3.3% (n=1)	0% (n=0)	43.3% (n=13)	26.7% (n=8)	0% (n=0)	56.7% (n=17)	30% (n=9)

[a] Data is compared for the same individuals. Week 8: n = 65. Week 24, n = 67.
[b] Data is compared for the same individuals. Week 8: n = 65. Week 24, n = 64.
[c] Data is compared for the same individuals; n = 30 at all time points.

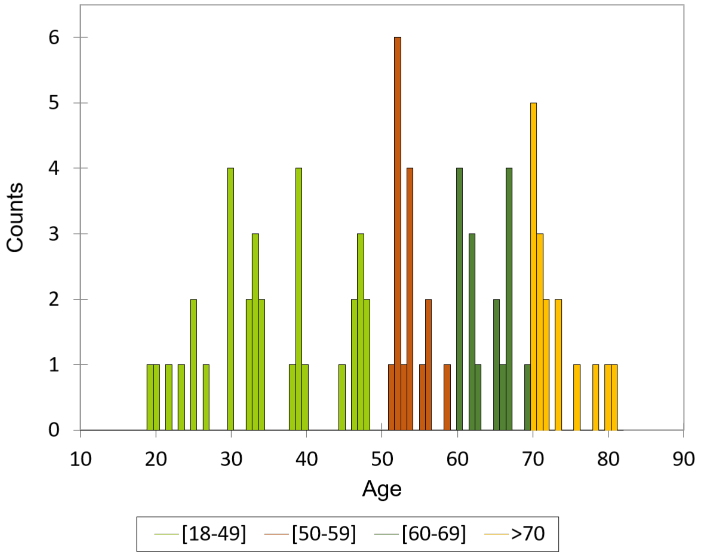

Figure S1. Age distribution of the study participants. The age stratification groups are shown in distinct colors.

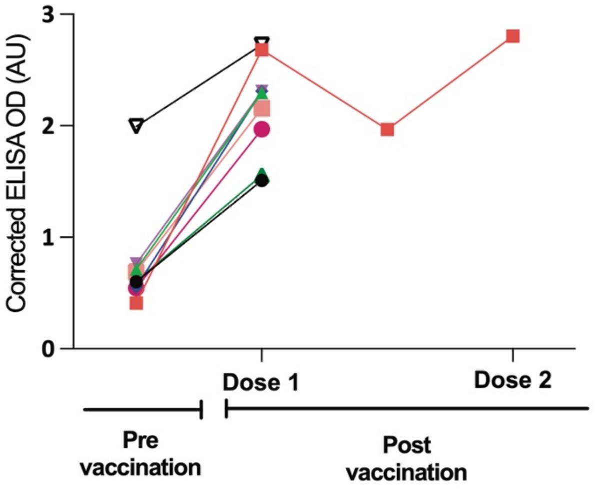

Figure S2. Immune response in SARS-CoV-2-positive patients pre- and post-vaccination. IgG ELISAs were conducted at the HQ site using serum samples from nine SARS-CoV-2-positive individuals who were vaccinated during the study; each is represented with a different color and symbol.
